# Unstable Expression of Commonly Used Reference Genes in Rat Pancreatic Islets Early after Isolation Affects Results of Gene Expression Studies

**DOI:** 10.1371/journal.pone.0152664

**Published:** 2016-04-01

**Authors:** Lucie Kosinová, Monika Cahová, Eva Fábryová, Irena Týcová, Tomáš Koblas, Ivan Leontovyč, František Saudek, Jan Kříž

**Affiliations:** 1 Laboratory of Pancreatic Islets, Center of Experimental Medicine, Institute for Clinical and Experimental Medicine, Prague, Czech Republic; 2 First Faculty of Medicine, Charles University in Prague, Prague, Czech Republic; 3 Department of Metabolism and Diabetes, Center of Experimental Medicine, Institute for Clinical and Experimental Medicine, Prague, Czech Republic; 4 Transplant Laboratory, Center of Experimental Medicine, Institute for Clinical and Experimental Medicine, Prague, Czech Republic; 5 Department of Diabetes, Center of Diabetes, Institute for Clinical and Experimental Medicine, Prague, Czech Republic; Communauté d'Universités et dÉtablissements Lille Nord de France, FRANCE

## Abstract

The use of RT-qPCR provides a powerful tool for gene expression studies; however, the proper interpretation of the obtained data is crucially dependent on accurate normalization based on stable reference genes. Recently, strong evidence has been shown indicating that the expression of many commonly used reference genes may vary significantly due to diverse experimental conditions. The isolation of pancreatic islets is a complicated procedure which creates severe mechanical and metabolic stress leading possibly to cellular damage and alteration of gene expression. Despite of this, freshly isolated islets frequently serve as a control in various gene expression and intervention studies. The aim of our study was to determine expression of 16 candidate reference genes and one gene of interest (F3) in isolated rat pancreatic islets during short-term cultivation in order to find a suitable endogenous control for gene expression studies. We compared the expression stability of the most commonly used reference genes and evaluated the reliability of relative and absolute quantification using RT-qPCR during 0–120 hrs after isolation. In freshly isolated islets, the expression of all tested genes was markedly depressed and it increased several times throughout the first 48 hrs of cultivation. We observed significant variability among samples at 0 and 24 hrs but substantial stabilization from 48 hrs onwards. During the first 48 hrs, relative quantification failed to reflect the real changes in respective mRNA concentrations while in the interval 48–120 hrs, the relative expression generally paralleled the results determined by absolute quantification. Thus, our data call into question the suitability of relative quantification for gene expression analysis in pancreatic islets during the first 48 hrs of cultivation, as the results may be significantly affected by unstable expression of reference genes. However, this method could provide reliable information from 48 hrs onwards.

## Introduction

Pancreatic islet transplantation into the portal vein of diabetic recipients represents a promising therapeutic alternative for the treatment of insulin-dependent diabetes mellitus since its re-launch by the Edmonton group in the year 2000 [1]. Despite the method has been considerably improved, its overall efficiency still needs to be enhanced. One of the possible approaches is to support islet viability during the isolation procedure and over subsequent steps by short-term silencing of specific genes, i.e. genes involved in apoptotic pathways, in coagulation, etc. This process requires manipulation and *in vitro* cultivation of isolated islets for several hours or days, whereby the precise quantification of target gene expression during all phases of islet isolation, preservation, and transplantation is the ultimate condition of success.

Quantitative real-time reverse transcription polymerase chain reaction (RT-qPCR) is a well-established method for quantifying mRNA in biological samples. Its benefits include high sensitivity, a large dynamic range, and the potential for high-throughput and accurate expression profiling of selected genes. Despite being a powerful technique, RT-qPCR is an indirect method prone to errors that are easily introduced through a number of steps during the experiment (sample handling, amount of starting material, RNA extraction, nucleic acid quality, enzymatic efficiencies, primer quality and characteristics), thereby increasing the risk of misinterpreting results [2,3]. Therefore, reliable normalization is required for the accurate determination of gene expression level with minimal experimental error. The most common method for normalizing cellular mRNA data is the use of reference genes (RGs) as internal controls; however, it is essential that the expression of selected gene(s) is stable and not affected by the experimental conditions [2–5]. Furthermore, an adequate control sample is necessary to estimate any changes or shifts in gene expression related to experimental interventions.

Isolation of pancreatic islets from exocrine tissue is complicated procedure which is critical for the survival of endocrine cells. Nevertheless, this process also exerts severe mechanical and metabolic stress on the islets and can lead to cellular damage and alteration of gene expression. In line with this, Marselli et al. [6] report in their study that immediately after isolation, 4560 genes were up-regulated and 1226 genes down-regulated in isolated islets compared with data obtained by microdissection from intact pancreatic tissue. Negi et al. [7] report that in freshly isolated islets, genes involved in mRNA catabolism are highly (21.9-fold) up-regulated, while genes associated with transcription are down-regulated (2.5-fold). Despite of this, both freshly isolated rodent and human islets frequently serve as controls in various gene expression and intervention studies, irrespective of the possible changes in gene expression due to the isolation stress [8–11].

Some conventional RGs, such as 18S rRNA, GAPDH, and β-actin, are classically considered as constitutively expressed in different tissues and often automatically used as normalizers because of their robust expression. However, there is a growing body of evidence showing that expression levels of these “classic” RGs can vary extensively due to diverse experimental conditions [3–5], thus making their routine use as internal controls inadequate for precise quantitative normalization because of the large measurement error [3–5,12,13], particularly in pancreatic islets [11,14]. Thus, appropriate validation step is a crucial requirement for avoiding misinterpretations of study findings, since the RGs selection appears to be highly specific for a particular experimental model [2].

To our best knowledge, no complex comparison study of frequently used RGs is available for islet grafts as well as evaluation of RG expression stability in the early period after isolation. Hence, we determined gene expression of the 16 most commonly used RGs (18S rRNA, Actb, Arbp, B2m, Gapdh, Gusb, Hmbs, Hprt, Pgk1, Ppia, Ppib, Rplp2, Tbp, Tfrc, Ubc, and Ywhaz) [15,16] and one gene of interest (tissue factor, F3) in freshly isolated rat pancreatic islets and after 24, 48, 72, 96 and 120 hrs of cultivation, in order to compare gene expression stability and to identify reliable RGs suitable for gene expression quantification.

## Methods

### Isolation and cultivation of rat pancreatic islets

All experiments were performed on Brown Norway rats (male, ~300 g) purchased from Charles River Laboratories, Germany, and were approved by The Animal Care Committee of the Institute for Clinical and Experimental Medicine and Ministry of Health of Czech Republic (Permit Number: 34/2012; 83/2013). Animals were held according to the European Convention on Animal Care in conventional breeding facility with 12/12 hrs light/dark cycle and free access to food pellets and water. A total of 38 rats were used in this study. Pancreatic islets were isolated using collagenase digestion followed by separation in a discontinuous density gradient, as previously described [17]. Briefly, pancreata of deeply anesthetized rats (Narketan/Dexdomitor 4:1, 0.065 ml/100 g b. wt.) were cannulated through the bile duct and filled with 15 ml of collagenase solution (Sevapharma, 1 mg/ml). After excision of pancreas, animals were sacrificed by exsanguinations. Excised pancreata were incubated for 18 min at 37°C with moderate shaking. Collagenase digestion was stopped by several cold Hank’s Balanced Salt Solution (supplemented with 1% FBS) washing steps and the tissue suspension was filtered through a 500 μm mesh. Pancreatic islets were separated using Ficoll (Sigma Aldrich) discontinuous density gradient (1.108 g/ml, 1.096 g/ml, 1.069 g/ml, 1.037 g/ml) yielding in 600–700 purified islets per rat. Isolated islets were harvested immediately after isolation or cultivated in CMRL-based culture medium supplemented with 10% FBS, 10 mM HEPES, 2 mM Glutamax, 100 U/ml Penicillin, and 100 μg/ml Streptomycin. Islets were harvested after 24, 48, 72, 96 and 120 hrs of cultivation and also in the period 0–24 hrs every 3 hrs. Samples of hand-picked islets free of exocrine tissue were frozen in liquid nitrogen and stored at -80°C until the RNA isolation. Pooled samples including islets of all sizes were used, i.e. islets with more than 150 um in diameter were not excluded from the study. The representative sample of isolated islets is shown on S1 Fig. Viability of isolated pancreatic islets was assessed using propidium iodide/acridine orange staining and ranged from 85 to 95% throughout the whole experiment. The insulin content in different time points of cultivation was proved by dithizone staining.

### Glucose-stimulated insulin secretion (GSIS) test

The ability of stimulated insulin secretion of freshly isolated islets and after 6, 24, 48, 72, 96 and 120 hrs of cultivation was determined using glucose-stimulated insulin secretion test. Prior the assay, islets (20 islets per well) were placed on polyester membranes with 8 μm pores (Transwell cell culture inserts, Corning) in six well plate with culture media. The GSIS assay medium consisted of Krebs-Ringer bicarbonate buffers equilibrated with 5% CO_2_ at 37°C, and supplemented with either 3 mM (low) or 22 mM (high) glucose. All incubations were carried on at 37°C and 5% CO_2_, in the volume of 4 ml. Islets were equilibrated for 15 minutes in low glucose GSIS assay medium. Then, glucose-stimulated insulin secretion was tested over 3 x 60 min at three subsequent concentrations of glucose (low, high, and low again). After each incubation period, the insulin content in every well was assessed by radioimmunoassay method (Insulin Coated Tube RIA Kit, MP Biomedicals). After the assay, islets were washed with PBS, collected and lyzed using proteinase K, EDTA and SDS (Sigma Aldrich). Total DNA content in each islet aliquot was determined using dsDNA specific assay, PicoGreen kit (Invitrogen).

### RNA isolation and reverse transcription

RNA was isolated using RNeasy Plus Mini Kit (Qiagen) including a column for elimination of genomic DNA. RNA concentration was determined using Qubit RNA HS Assay Kit (Life Technologies). RNA purity was assessed using NanoDrop 2000 UV-Vis Spectrophotometer as the 260 nm/280 nm absorbance ratio. In order to prove the RNA quality, the RNA Integrity Number (RIN) was measured in a separate experiment using Agilent RNA 600 Nano Kit (Agilent Technologies). Based on electrophoretic separation, this method provides sizing, quantification and quality control of RNA. Also, the average RNA yield for one islet was calculated in different time points of cultivation. The fixed amount of RNA (250 ng) was reversely transcribed to cDNA with High-Capacity RNA-to-cDNA Kit (Life Technologies) according to manufacturer´s instructions and stored at -20°C until the gene expression analysis.

### RT-qPCR

In the first experiment, the expression of 16 candidate RGs (18S rRNA, Actb, Arbp, B2m, Gapdh, Gusb, Hmbs, Hprt, Pgk1, Ppia, Ppib, Rplp2, Tbp, Tfrc, Ubc, Ywhaz) normalized to the same RNA input was determined using TaqMan^®^ Rat Endogenous Control Array 384-well micro fluidic cards on AbiPrism 7900 (Life Technologies). Full names, IDs, function and location of all genes can be found in S1 Table. In the second experiment, absolute quantification of Gapdh, Ppia, and F3 was performed. Specific primers for these genes (F3: 5′-GATAAAGACAGTGACCAGGAACA-3′, 5′-CTAACCACAAGAGCCCAGAA-3′; Gapdh: 5′-GTAACCAGGCGTCCGATAC-3′, 5′-TCTCTGCTCCTCCCTGTTC-3′; Ppia 5′-CCATTATGGCGTGTGAAGTC-3′, 5′-GCAGACAAAGTTCCAAAGACAG-3′) (IDT) were used to amplify the defined segment of the respective mRNA. Amplified segments were separated on 2% agarose gel and purified with QIAquick Gel Extraction Kit (Qiagen). DNA concentration was determined using Qubit DNA Assay Kit (Life Technologies). Purified fragments were used to construct the calibration curves. As a template for segment amplification, total RNA isolated from islets after 72 hrs of cultivation was used. Absolute quantification of F3, Gapdh and Ppia gene expression was performed on ViiA^™^ 7 Real-Time PCR System (Applied Biosystems) using PrimeTime^®^ qPCR probe-based gene expression assays (IDT).

### Data analysis and statistics

Gene expression data were analyzed using RQ Manager and ViiA^™^ software. Absolute cDNA quantity was calculated from the appropriate calibration curve. The stability of individual RGs was evaluated using GeNorm software, a Microsoft Excel program available at https://genorm.cmgg.be/. Friedman’s Two Way ANOVA and multiple comparison method were used to analyze the differences between gene expression at different time points (0, 24, 48, 72, 96 and 120 hrs) of cultivation. Differences were considered statistically significant at the level of p < 0.05.

## Results

### Analysis of RNA quality

**Fig 1:**
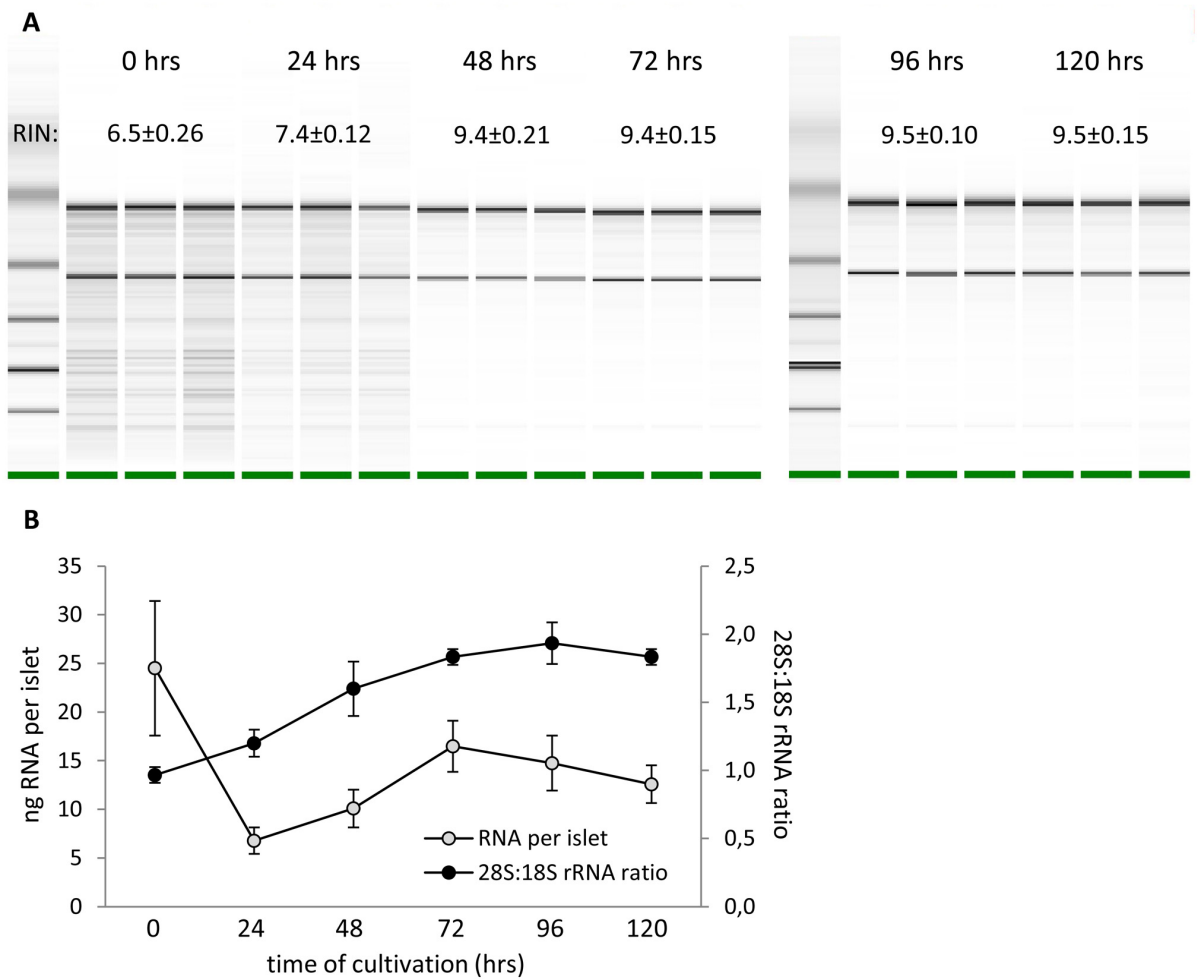
RNA integrity and quantity. A: Electropherograms of RNA isolated from islets at different time of cultivation. RIN = RNA integrity number. B: RNA quantity per one islet and 28S:18S rRNA ratio in the same RNA samples.

RNA purity determined as the 260 nm/280 nm absorbance ratio was ≥ 1.97 for all samples. RNA integrity was assessed as the RNA Integrity Number (RIN). Ranging from 6.5 to 9.5 for all RNA samples, RIN showed good quality of RNA (with 1 being the most degraded sample and 10 being the most intact). Nevertheless, RNA isolated from freshly prepared islets and islets cultivated for 24 hrs exhibited partial fragmentation (RIN 6.5±0.26 and 7.4±0.12, respectively). In contrast, when culturing islets for 48 hrs or more, the RIN was close to 10 (RIN ≥ 9.4) what indicates nearly intact RNA. The RNA electropherograms are shown in Fig 1A. A detailed quantification of individual electropherogram band densities is shown in S2 Fig. The 28S:18S rRNA ratio, another parameter reflecting RNA integrity, increased from 1.0 at time 0 hrs to 1.9 at 96 hrs and stabilized at 1.8 at 120 hrs of cultivation. We found the highest RNA yield per islet immediately after isolation (T = 0 hrs) followed by significant drop during next 24 hrs and consequent elevation up to 72 hrs of cultivation (Fig 1B).

### Metabolic characterization of islets

**Fig 2:**
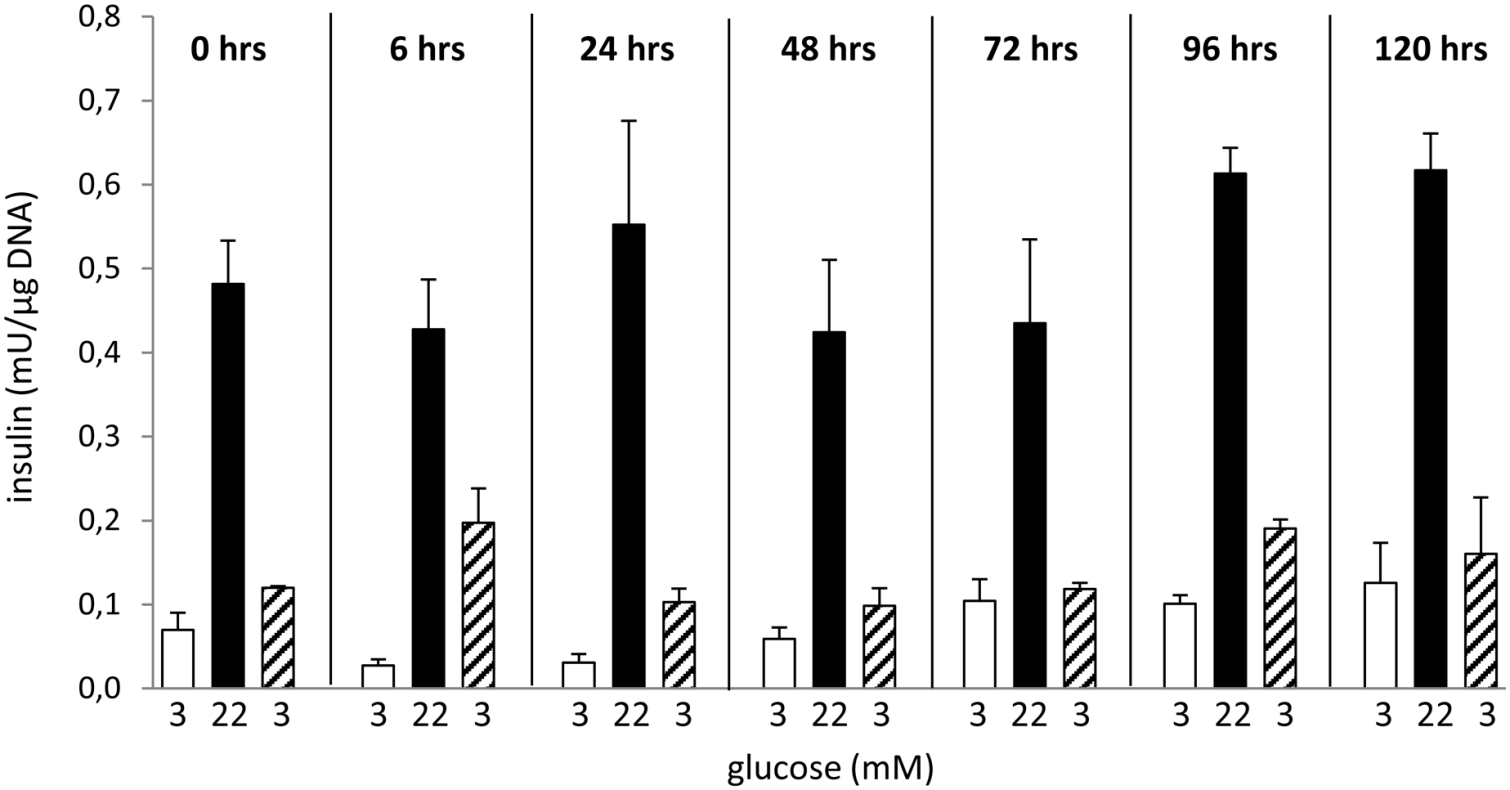
Effect of the length of cultivation on glucose-stimulated insulin secretion (GSIS) of pancreatic islets *in vitro*. Glucose-stimulated insulin secretion was tested at three subsequent concentrations of glucose (3 mM, 22 mM, and 3 mM again). Data are given as a mean ± SD, n = 3.

**Fig 3:**
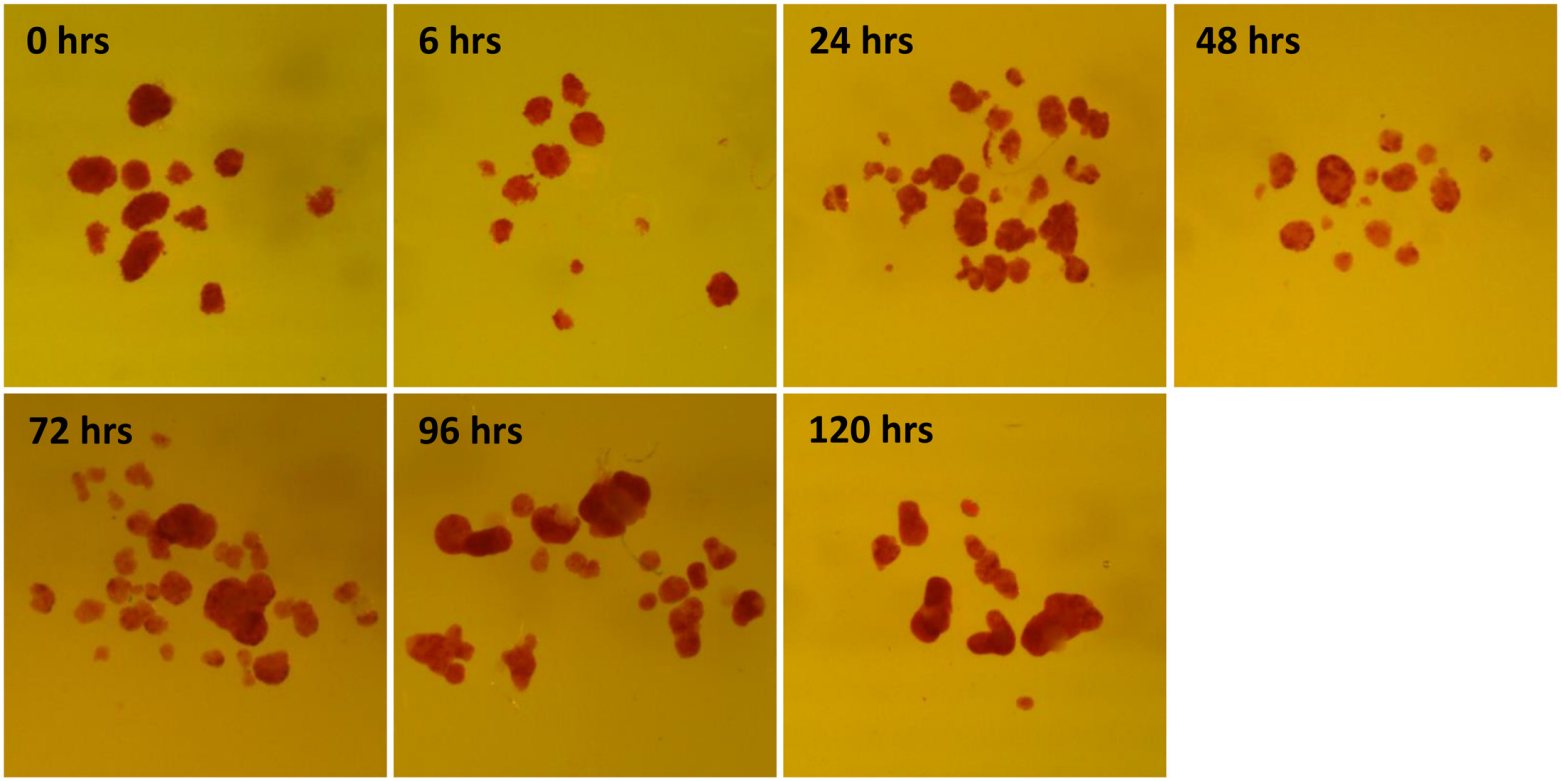
Dithizone staining of islets cultivated for different time periods. Magnification 15×.

The metabolic state of isolated islets in various time points of cultivation was evaluated according to their capacity to respond to glucose stimulation *in vitro* (Fig 2) and by the verification of insulin presence by dithizone staining (Fig 3). The ability of the isolated islets to increase insulin secretion after glucose challenge did not significantly change throughout the 120 hrs cultivation period. The stimulatory index (ratio of insulin secretion in basal and stimulated conditions) was highest at 6 hrs (16±2.5) and 24 hrs after isolation (19±5.3). Nevertheless, from the absolute values (Fig 2) it is clear, that this value reflects rather low basal than elevated glucose-stimulated insulin secretion. Since 48 hrs of cultivation onwards the stimulation index was quite stable (6.1±2.0). The well-preserved metabolic flexibility of cultivated islets is demonstrated by their ability to decrease insulin secretion when transferred back to low glucose medium. During the cultivation, we did not observe any attenuation of dithizone staining intensity what confirmed that insulin production was not compromised.

### Expression profiling of candidate reference genes

**Fig 4:**
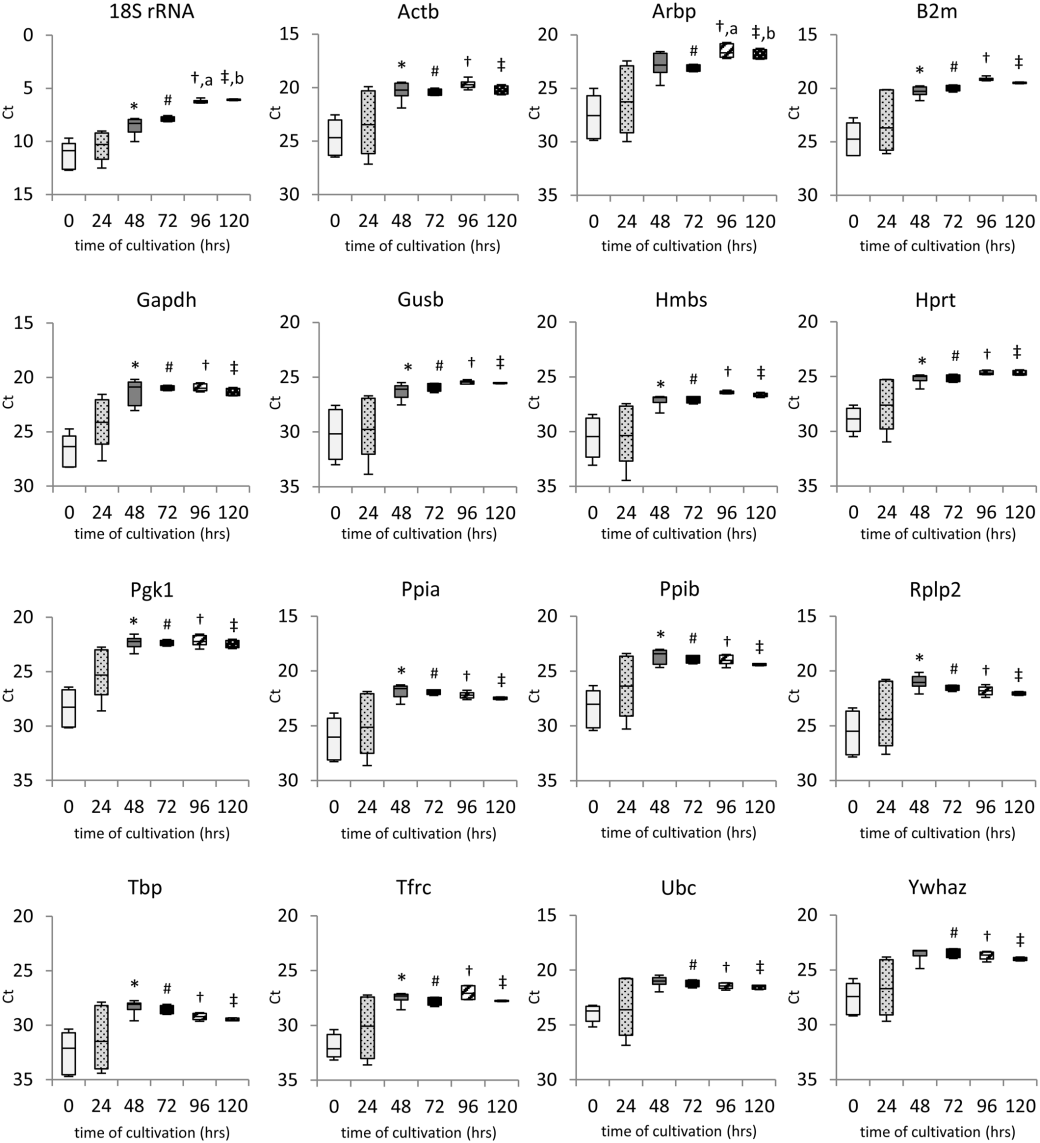
Expression of candidate reference genes during cultivation. Ct of individual candidate genes are shown as medians (lines), 25th percentile to the 75th percentile (boxes) and as ranges (whiskers) immediately after isolation and at 24, 48, 72, 96 and 120 hrs of cultivation. Data are based on at least 6 independent experiments. Statistical significance of the differences in Ct values was evaluated using Freidman’s Two Way ANOVA and multiple comparison method; *p < 0.05 48 vs. 0 hrs; ^#^ p < 0.05 72 vs. 0 hrs; †p < 0.05 96 vs. 0 hrs; ‡ p < 0.05 120 vs. 0 hrs; ^a^ p < 0.05 96 vs. 48 hrs; ^b^ p < 0.05 120 vs. 48 hrs.

The first requirement for RG is its stable expression. To examine the impact of the isolation procedure and subsequent cultivation on the candidate RGs, their expression was measured in freshly isolated islets (0 hrs) or islets cultivated *in vitro* for 24, 48, 72, 96 and 120 hrs. As shown in Fig 4, none of the genes tested in our study met the stability requirements over the whole cultivation period. In all cases, the specific mRNA level increased rapidly in the first 48 hrs of cultivation whereat it stabilized. During first 72 hrs of cultivation, it increased 15 times on average (from 5.8-fold for 18S rRNA to 43.1-fold for Pgk1) while about 90% of the increase occurred during the first 48 hrs of cultivation. In contrast, from 72 to 120 hrs of cultivation, the specific mRNA level remained relatively stable with fold change of about 1.5 on average. The only significant changes in the interval 48–120 hrs were found in the expression of 18S rRNA and Arbp gene. Furthermore, variability which was very high among samples at 0 and 24 hrs decreased significantly after 48 hrs of cultivation.

**Fig 5:**
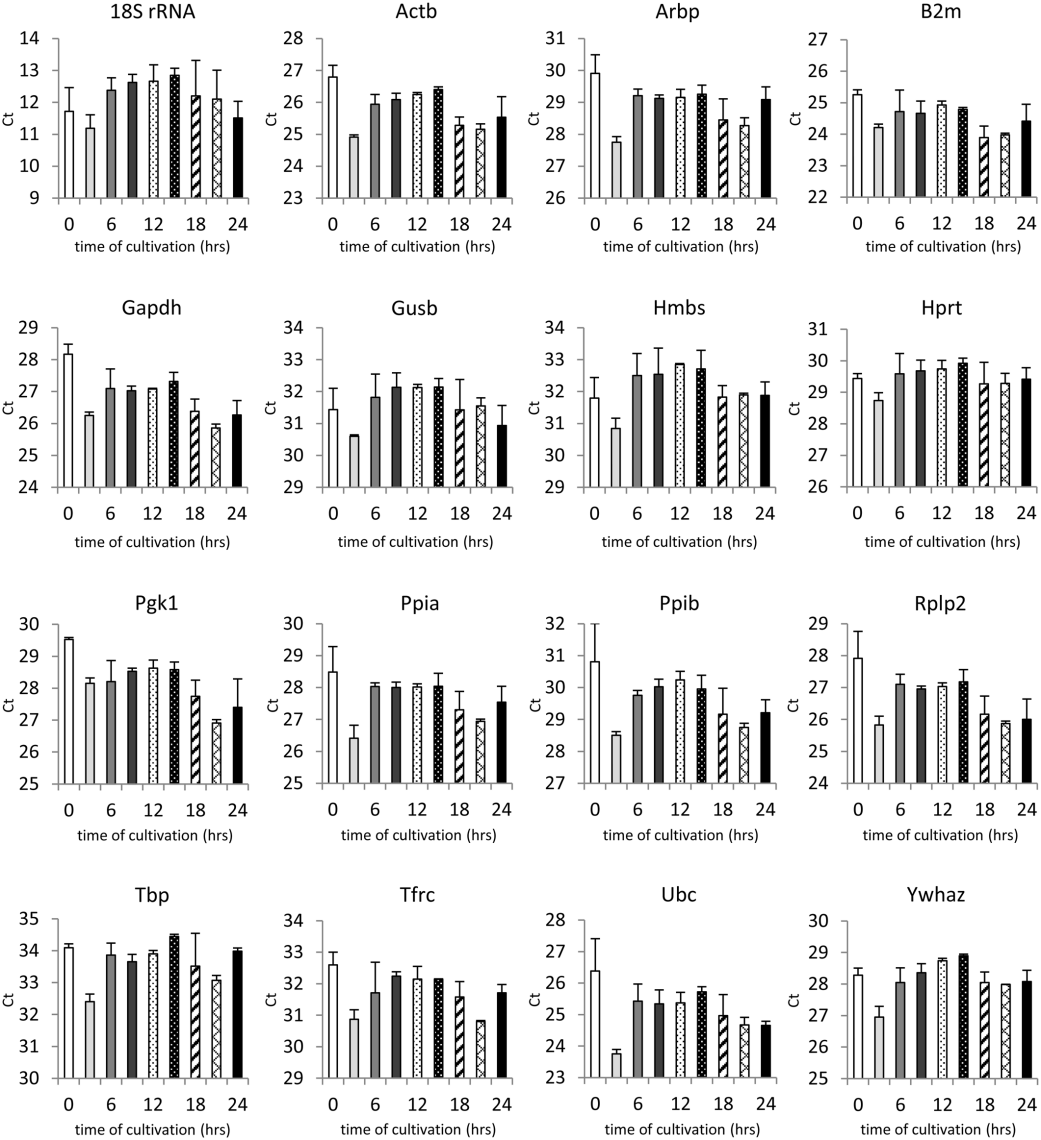
Expression of candidate reference genes during the first 24 hours of cultivation. Ct of individual genes are shown as mean ± S.D., n = 6. Data are based on two independent experiments.

In order to learn more about the course of events during the early phase of cultivation (0–24 hrs), we determined the RGs’ expression every 3 hrs (Fig 5). For all genes, the expression pattern shared some common features. In general, the highest expression rate was observed at 3 hrs after isolation. Then the expression rate decreased and was relatively stable between 6 and 15 hrs after isolation. The time interval from 18 to 24 hrs was characterized by significant variability both among genes and individual time points.

**Fig 6:**
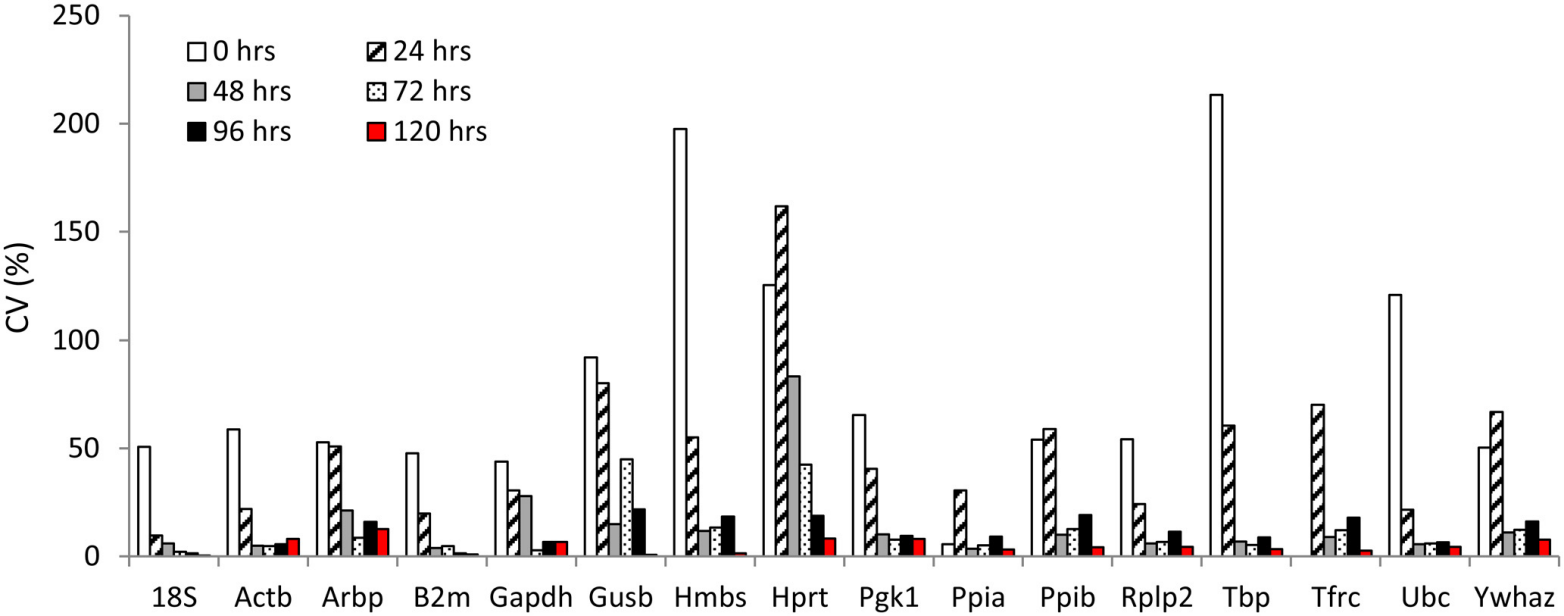
Coefficient of variation (%) ΔCt_(GOI-RG)_ at different time points of cultivation. Data are based on at least 6 independent experiments.

The changing expression of RGs throughout the cultivation period may not necessarily disqualify them as normalizers for relative quantification at specific time point of cultivation, provided that ΔCt_(GOI-RG)_ is nearly constant for one time point and one particular gene of interest (GOI)/RG combination. In order to test this presumption, we calculated ΔCt_(GOI-RG)_ for different time points of cultivation for all candidate RGs and F3 (GOI) mRNA (Fig 6). At the time after isolation and after the first 24 hrs of cultivation, ΔCt_(GOI-RG)_ was highly variable and the coefficient of variation (CV) oscillated in hundreds of percent for all genes. In contrast, from 48 hrs onwards, expression of both GOI and RGs stabilized and ΔCt_(GOI-RG)_ remained constant with CV not exceeding units of percent. Taken together, these data indicate that gene expression is significantly disturbed by the isolation procedure, at least at transcription level, and its stabilization occurs only after 48 hrs of cultivation.

### Stability of reference genes with respect to individual stages of pancreatic islets cultivation

**Fig 7:**
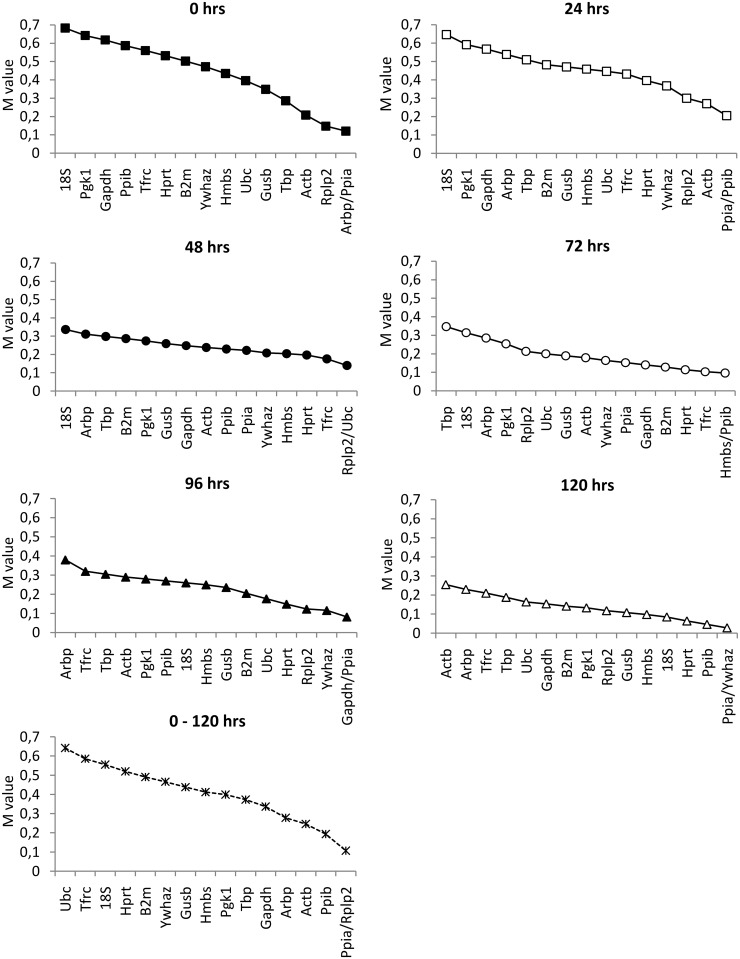
Expression stability values of candidate RGs at different phases of cultivation determined by GeNorm. The *M* value represents an average stability measure of each possible combination of a particular RG with all other genes in the multiplex. The lower the *M* value of a given gene, the more consistent its expression relative to other genes in the multiplex.

There are several methods used for the identification of the most stable gene combinations. The GeNorm algorithm enables the gene stability measure *M* to be calculated as the average pairwise variation (V) between one particular gene and all other candidate RGs. Genes with the lowest *M* value have the most stable expression [18]. Fig 7 shows the *M* values for individual genes defined by GeNorm at 0, 24, 48,72, 96 and 120 hrs of cultivation and over the whole cultivation period (0–120 hrs). Rather surprisingly, all calculated *M* values lay well below the arbitrarily suggested cut-off value (M = 1.5) for unstable genes. On the other hand, we did not find any set of RGs that would provide an equal rate of expression stability at all time points of cultivation. Pairwise variation analysis did not indicate the beneficial effect of including more than two RGs, as the *V* value was well below the recommended cut-off value of 0.15 (data not shown).

**Fig 8:**
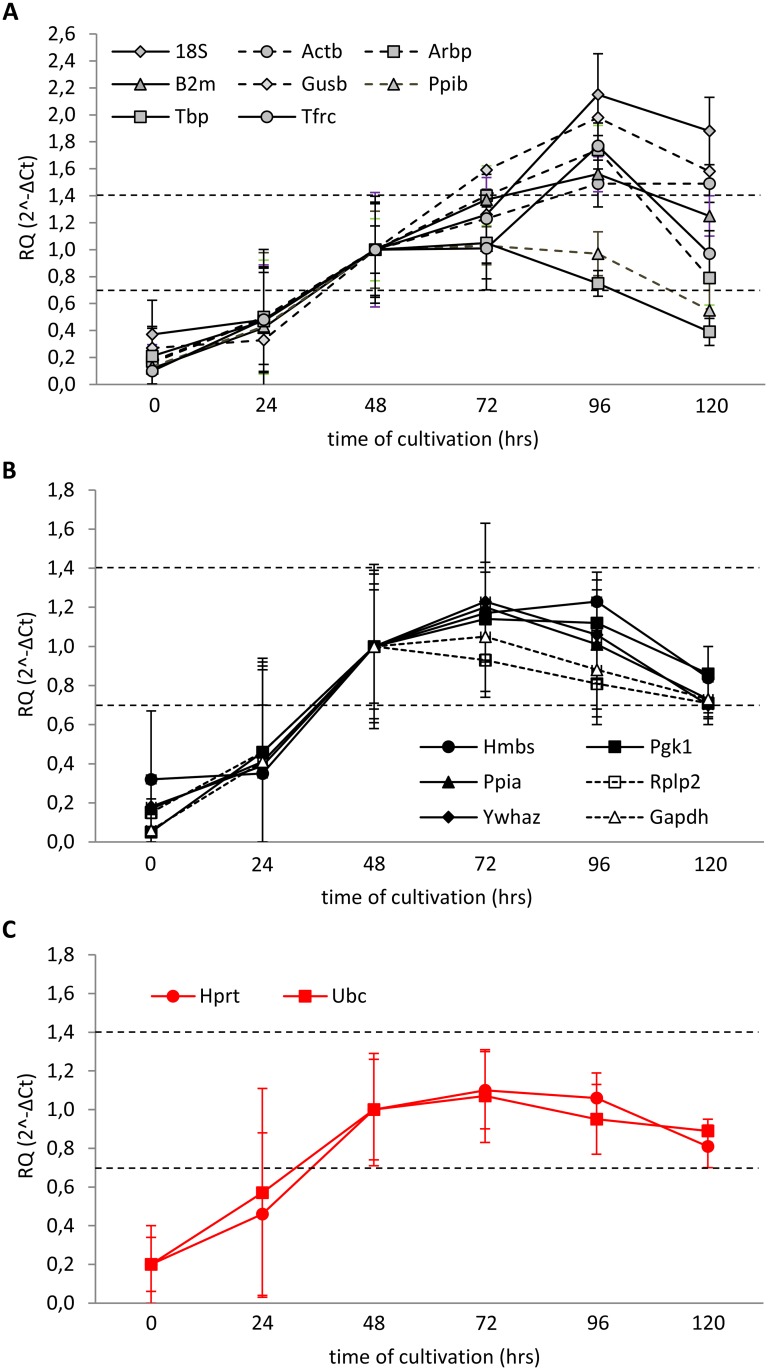
Expression stability of candidate RGs at different phases of cultivation evaluated according to the ±0.5 ΔCt rule. Data are expressed as means ± SD, n = 6. Dashed lines indicate the interval of 0.7-1.4-fold change compared with the expression at 48 hrs.

Another approach has been published by Gorzelniak et al. [19]. According to their guideline, the expression of proper RG should not differ by more than half of the cycle (ΔCt ≤ 0.5) between control and experimental samples. Employing this criterion, we evaluated the suitability of all 16 candidate RGs. In order to make the statistical evaluation possible, we converted ΔCt data to “fold changes” using the 2^-ΔCt^ method. In this case, ΔCt of +0.5 and -0.5 are equivalent to 0.7 and 1.4-fold changes in relative gene expression, respectively [20]. Because gene expression in our experiment seemed to stabilize at 48 hrs, we chose average expression of the respective candidate RGs at 48 hrs as a calibrator. None of the tested genes met the ±0.5 ΔCt rule in freshly isolated islets or at 24 hrs after isolation (Fig 8A, 8B and 8C). When considering only the period from 48 to 120 hrs of cultivation, eight of the genes (18S, Actb, Arbp, B2m, Gusb, Ppib, Tbp, Tfrc) (Fig 8A) were out of the limit in at least one of cultivation times evaluated. The average expression of six genes (Gapdh, Hmbs, Pgk1, Ppia, Rplp2, Ywhaz) met the ±0.5 ΔCt rule during the interval 48–120 hrs but due to the variance their expression in some individual samples exceeded 0.7 or 1.4-fold change borders (Fig 8B). The only two genes that absolutely complied with the ±0.5 ΔCt rule were Hprt and Ubc (Fig 8C).

### Comparison of relative vs. absolute quantification of gene expression in isolated pancreatic islets

**Fig 9:**
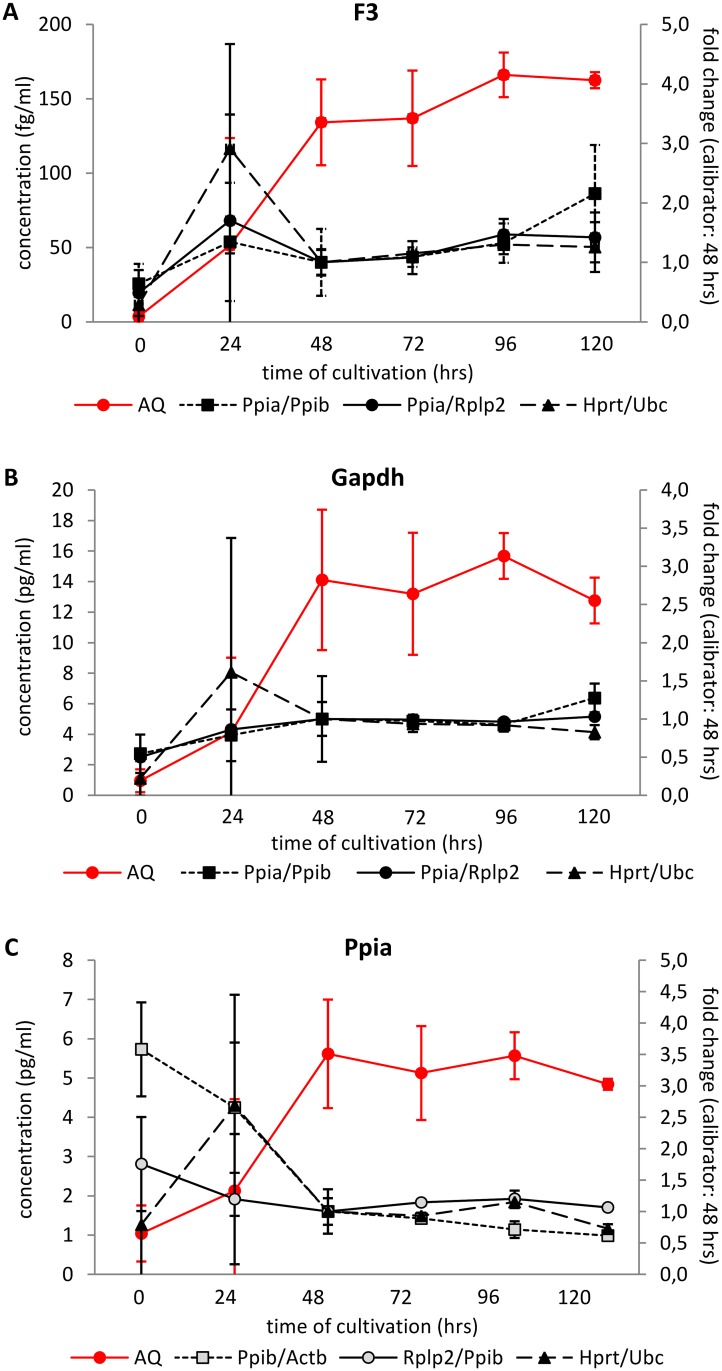
Relative and absolute quantification of F3, Gapdh and Ppia in isolated pancreatic islets during *in vitro* cultivation. Relative quantification was calculated using the 2^-ΔΔCt^ method related to time 48 hrs; absolute expression was determined using a calibration curve constructed for each specific transcript. RG pairs were chosen either by GeNorm, calculated for expression at 24 hrs only (Ppia/Ppib), or for the whole interval 0–120 hrs (Rplp2/Ppia) or by the ±0.5 ΔCt rule (Hprt/Ubc). When Ppia was evaluated as GOI, we used the next most stable reference gene identified by GeNorm (24 hrs: Ppib/Actb; 0–120 hrs: Rplp2/Ppib).

In order to test the plausibility of relative quantification of gene expression at different time points during pancreatic islet cultivation, we employed the 2^-ΔΔCt^ method when the expression at 48 hrs was set as calibrator and calculated the expression of three genes appointed as “GOIs” using three RG combination: 1) most stable pair identified by GeNorm at 24 hrs; 2) most stable pair identified by GeNorm over the whole cultivation period (0–120 hrs) and 3) RG pair identified by the ±0.5 ΔCt rule. We compared these results with data obtained by the absolute quantification method which determined the respective mRNA concentration using a calibration curve constructed for the specific transcript (Fig 9A, 9B and 9C). We chose the following genes of interest: Gapdh, one of the most widely used RGs; Ppia, identified as one of the most stable genes in our experimental setting; and F3 (tissue factor), which is referred to increase its expression during the *in vitro* cultivation [21].

According to the absolute quantification method, we observed a similar pattern of expression for all three GOIs—their expression was very low at the time of isolation and then increased up to 48 hrs of cultivation after which it remained relatively stable. In contrast to this, the results of relative quantification failed to reflect the changes in respective mRNA concentrations determined using the absolute quantification method during the first 48 hrs for all three GOIs irrespective to the RG pair chosen. In the interval 48–120 hrs, the expression of GOIs determined by relative expression generally paralleled their expression assessed by absolute quantification. Both methods identified enormous variance at 24 hrs of cultivation.

These data confirm our presumption that due to the unstable expression of most of the genes during the initial phase of *in vitro* cultivation, relative quantification of mRNA expression is not a suitable method as it does not reflect the real mRNA concentrations during the first 48 hrs of cultivation.

## Discussion

Our study focused on the identification and validation of a suitable set of RGs for use in gene expression normalization during short-term cultivation of isolated rat pancreatic islets. We found that none of the 16 candidate RGs met the stability criteria (ΔCt ≤ ±0.5) throughout the whole 120 hrs cultivation period. Our main findings can be summarized as follows: first, immediately after isolation, the specific mRNAs content was many times lower in comparison with all subsequent phases of cultivation and RNA exhibited considerable degradation; second, during the recovery phase which lasted for subsequent 48 hrs, transcription of all tested genes increased but it was extremely variable among samples which were supposed to be homogenous; third, from 48 hrs of cultivation onwards, RNA integrity increased and expression levels of all tested genes stabilized with minimal variability among samples; finally, none of the candidate RGs met the acceptable fluctuation criteria (ΔCt ≤ ±0.5) during the first 48 hrs of cultivation. Taken together, our data suggest that normalization during the first stages of pancreatic islet cultivation is problematic and raise questions as to the suitability of gene expression studies and particularly of the relative quantification method under these conditions.

RT-qPCR is a widely-used method for quantitative determination of gene expression in biological samples; however, it is also quite prone to a number of imprecision and technical errors. These problems could be overcome by normalization, i.e. including an invariant endogenous control in the assay to correct both sample-to-sample variations in RT-qPCR efficiency and errors in sample quantification. However, it is essential that expression of the selected endogenous control (i.e. RG) is stable and not affected by the experimental conditions used in the study under investigation [1,4].

In our experiment, we found that immediately after pancreatic islet isolation, expression of all tested candidate RGs was significantly down-regulated when compared to that at 48 hrs of cultivation (5- to 40-fold, 13,5-fold in average). This is probably due to the ischemic, mechanical, osmotic, and oxidative stress to which pancreatic islets are exposed during the isolation procedure [22]. As previously published, during the process of isolation, islet cells undergo profound changes in structure and function resulting in beta-cell apoptosis [23], whether as a consequence of hypoxia, disruption of the native islet cell microenvironment or lack of growth factors [23–27]. Furthermore, the isolation procedure potently recruits some of stress signaling pathways; however, culturing of islets for 48 hrs after isolation allows for activated pathways to return to background levels [28].

In order to verify the quality of RNA isolated from islets harvested at different time points of cultivation, we measured integrity and possible degradation of RNA as it may also influence the results of RT-qPCR analysis. According to RIN and the corresponding electropherograms (Fig 1A and S2 Fig), RNA obtained from freshly isolated islets and from islets cultivated for 24 hrs exhibits partial fragmentation (RIN 6.5±0.26 and 7.4±0.12, respectively) while the RNA from islets cultivated for 48 hrs or more is nearly intact (RIN ≥ 9.4). 28S:18S rRNA ratio, another parameter reflecting the degree of RNA degradation, was low in freshly isolated islets (1.0) but then rose up and got stabilized (1.9 and 1.8) after 96 and 120 hrs of cultivation, respectively. Finally, the amount of RNA obtained from one islet was highest in freshly isolated islets, then it dropped rapidly, reached the minimal value after 24 hrs and then increased slowly again (Fig 1B). All these observations could be explained by launching of degradation processes through the stress to which islets are exposed during the isolation procedure [28]. These mechanisms probably lead to general destruction of RNA resulting in rapid decrease in RNA content during the first 24 hrs after isolation. In tissue culture, as the degradation and stress signaling pathways are not stimulated anymore [28], the RNA content start to rise and the islet condition improves. After 48 hrs of cultivation, there is no evidence of RNA degradation (Fig 1). Nevertheless, the initial RNA content per one islet is not reached again, probably due to natural cell death, eventually also due to central necrosis of some islets during cultivation. For these reasons, along with the time of cultivation, the number of islet cells naturally decreases which means that the RNA content per one islet drops as well. Interestingly, the main metabolic function of the islets, the ability to respond to glucose stimulation by the increased insulin secretion, was not compromised at any time point of cultivation. The intactness and the presence of insulin in the islets throughout the cultivation were evidenced by dithizone staining. Taken together, our data indicate that isolation stress affects particularly the RNA stability and/or synthesis while the main metabolic characteristics are unaffected.

According to our observations, during the first 48 hrs of cultivation, significant fluctuations in gene expression occurred, while from 48 hrs onwards, substantial stabilization took place. Particularly, 24 hrs after islet isolation, gene expression of all tested genes was extremely variable among the samples which were supposed to be homogenous. Also in the time period 0–24 hrs, the expression of all genes varied significantly both among times and genes. In contrast, after 48 hrs of cultivation, Ct values stabilized with minimal variability among samples, for most of the genes. This observation questions the biological relevance of gene expression studies during the early phase (0–48 hrs) of islet cultivation *in vitro*.

Several approaches have been developed for selection of a suitable internal control. The “empirical” approach is based on selecting genes with robust expression, i.e. Gapdh, B2m, or 18S rRNA, which have been successfully used in non- or semiquantitative methods, such as Northern blot where qualitative changes are measured [29]. Even though there is a wealth of evidence indicating that these genes are significantly affected by different conditions [2,7–12,30], they are still frequently used in gene expression studies carried out on isolated pancreatic islets [12,31]. In our experiment, some of these genes (e.g. 18S rRNA) were found to be quite unstable (Figs 4, 7 and 8).

Another approach is based on the precise evaluation of RG stability under particular experimental conditions. According to Gorzelniak [19], differences in ΔC_T_ between “experimental” and “control” sample for particular RG < 0.5 are caused by technical variance of the method and are likely to be reflected by the gene of interest and RG in the same manner. In contrast, ΔC_T_ values >1.0 reflect changes in gene expression levels and indicate that candidate RG expression is influenced by experimental conditions. In our study, ΔCt ranges from 2.3 to 5.3 during the first 48 hrs after isolation. Therefore, the use of any of 16 candidate RGs as normalizers for relative quantification of gene expression during the first 48 hrs of cultivation is inappropriate and would lead to erroneous results. However, after 48 hrs of cultivation, expression of eight of the potential RGs (Gapdh, Hmbs, Hprt, Pgk1, Ppia, Rplp2, Ubc, Ywhaz) stabilized with the ±0.5 ΔCt range. Although the expression level of some genes moved slightly up or down from 72 hrs onwards, the variability of expression among samples in one group decreased significantly (Figs 4 and 6).

The GeNorm method [18] was developed for determination of gene expression stability on the basis of non-normalized expression levels. The GeNorm algorithm allows gene-stability measure *M* to be calculated as the average pairwise variation (*V*) between the particular gene and all other candidate RGs. In contradiction to our findings, all calculated *M* values provided by GeNorm lay well below the arbitrarily suggested cut-off value (*M* = 1.5; with a lower value indicating increased gene stability), which means that all combinations of two tested genes should be stable enough to be used as internal controls. Nevertheless, as shown in Figs 4 and 8, expression of all candidate RGs significantly increased during the first 48 hrs of cultivation. This discrepancy could be explained by the mechanism used for GeNorm calculation, which evaluates the stability of each combination of two genes remaining in a multiplex after one-by-one exclusion according to a descending *M* value until only two genes with the most stable expression ratio remain. Due to the GeNorm’s pairwise comparison these cannot be further differentiated. Because the expression of all genes during the 48 hrs after isolation followed a similar pattern, the ratio between every two compared genes appeared to be stable. This may also explain why there was no need to include more than two RGs according to GeNorm’s pairwise variation analysis, since the calculated *V* value fell well below the recommended cut-off value of 0.15 (data not shown).

Finally, the limited suitability of relative quantification in isolated pancreatic islets during the first 48 hrs of cultivation is illustrated if we compare the results of relative and absolute quantification of the three genes: F3, Gapdh, and Ppia (Fig 9). F3 gene encodes the protein of tissue factor which plays a key role in tissue engraftment when performing pancreatic islet transplantation. Tissue factor triggers the Instant Blood Mediated Inflammatory Reaction (known as IBMIR) immediately after pancreatic islet infusion into the portal vein blood, thus influencing the ratio of destroyed/engrafted islets. The increasing expression of F3 gene during tissue culture preservation of islets before transplantation was repeatedly reported. Gapdh gene was chosen as one of the most "popular" housekeeping genes used as normalizers in gene expression analysis. Ppia gene was selected as it seems to be one of the most stable genes in our experimental setting (Fig 7). Absolute quantification showed a similar expression pattern for all three genes. The gene copy number per sample was very low immediately after isolation, then increased over the next 48 hrs and remained relatively stable between 48 hrs and 120 hrs. In contrast to this, the results of relative quantification failed to reflect the changes in respective mRNA concentrations determined using the absolute quantification method during the first 48 hrs for all three GOIs irrespective to the RG pair chosen. In the interval 48–120 hrs, the expression of GOIs determined by relative quantification generally paralleled the expression assessed by absolute quantification. Better results were obtained when using RG pairs identified according to their expression during the whole cultivation period both by GeNorm or ±0.5 ΔCt rule than by RG pair chosen according to the expression at one particular time point, i.e. at 24 hrs.

In conclusion, we provide here evidence that during the first 48 hrs of isolated pancreatic islet cultivation, expression of commonly used RGs is unstable apparently due to the isolation stress which probably leads to nonspecific RNA destruction and activation of RNA degradation pathways. During this period, data obtained using relative quantification do not reflect actual changes in specific mRNA concentrations and related calculations can lead to misinterpretation of study findings. Therefore, we suggest that other methods rather than commonly used relative quantification should be used to determine gene expression in freshly isolated pancreatic islets or islets should be stabilized in tissue culture 48 hrs before gene expression studies. From 48 hrs onwards, relative quantification method can provide reliable information in isolated pancreatic islets. Our findings highlight the importance of RG validation step for every particular experimental setting.

## Supporting Information

S1 Fig Representative samples of dithizone stained islets at 0, 24, and 48 hrs after isolation. Magnification 15×.
(PDF)

S2 Fig Electropherograms of RNA samples isolated from islets at different time points of cultivation. (PDF)

S1 Table Official symbol, ID, full name, function and location of 16 candidate reference genes and the F3 gene. Available at http://www.ncbi.nlm.nih.gov/gene/.
(PDF)
