# Supplementary material for: Unstable Expression of Commonly Used Reference Genes in Rat Pancreatic Islets Early after Isolation Affects Results of Gene Expression Studies
Source: PLoS One. 2016 Apr 1;11(4):e0152664. doi: 10.1371/journal.pone.0152664 (PMC4817981; doi:10.1371/journal.pone.0152664)

S1 Fig: Representative samples of dithizone stained islets at 0, 24, and 48 hrs after isolation. Magnification 15×.

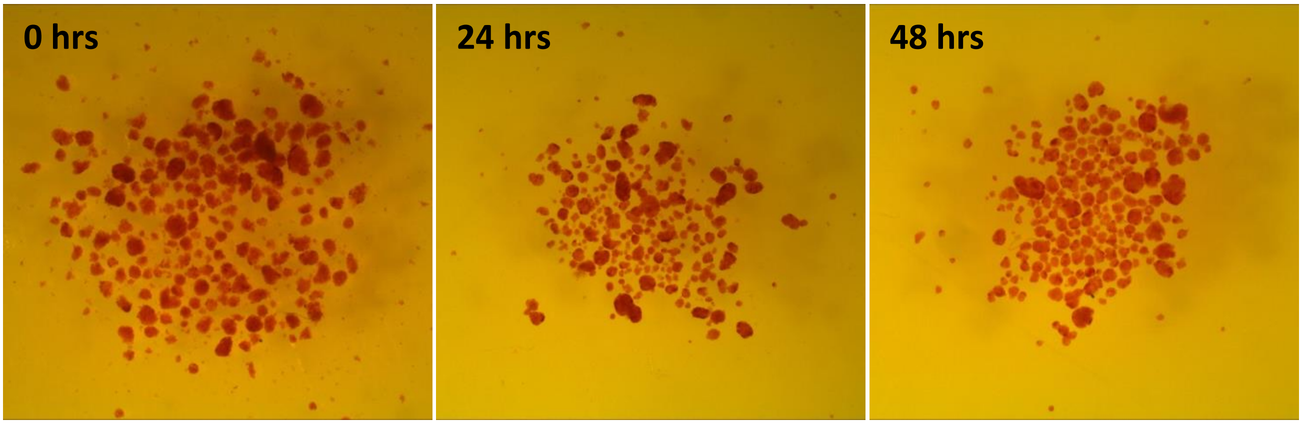

Supplement: S1 Fig — Magnification 15×. (PDF) [file pone.0152664.s001.pdf]
