# Supplementary material for: Unstable Expression of Commonly Used Reference Genes in Rat Pancreatic Islets Early after Isolation Affects Results of Gene Expression Studies
Source: PLoS One. 2016 Apr 1;11(4):e0152664. doi: 10.1371/journal.pone.0152664 (PMC4817981; doi:10.1371/journal.pone.0152664)
Supplement: S2 Fig — (PDF) [file pone.0152664.s002.pdf]

S2 Fig: Electropherograms of RNA samples isolated from islets at different time points of cultivation.

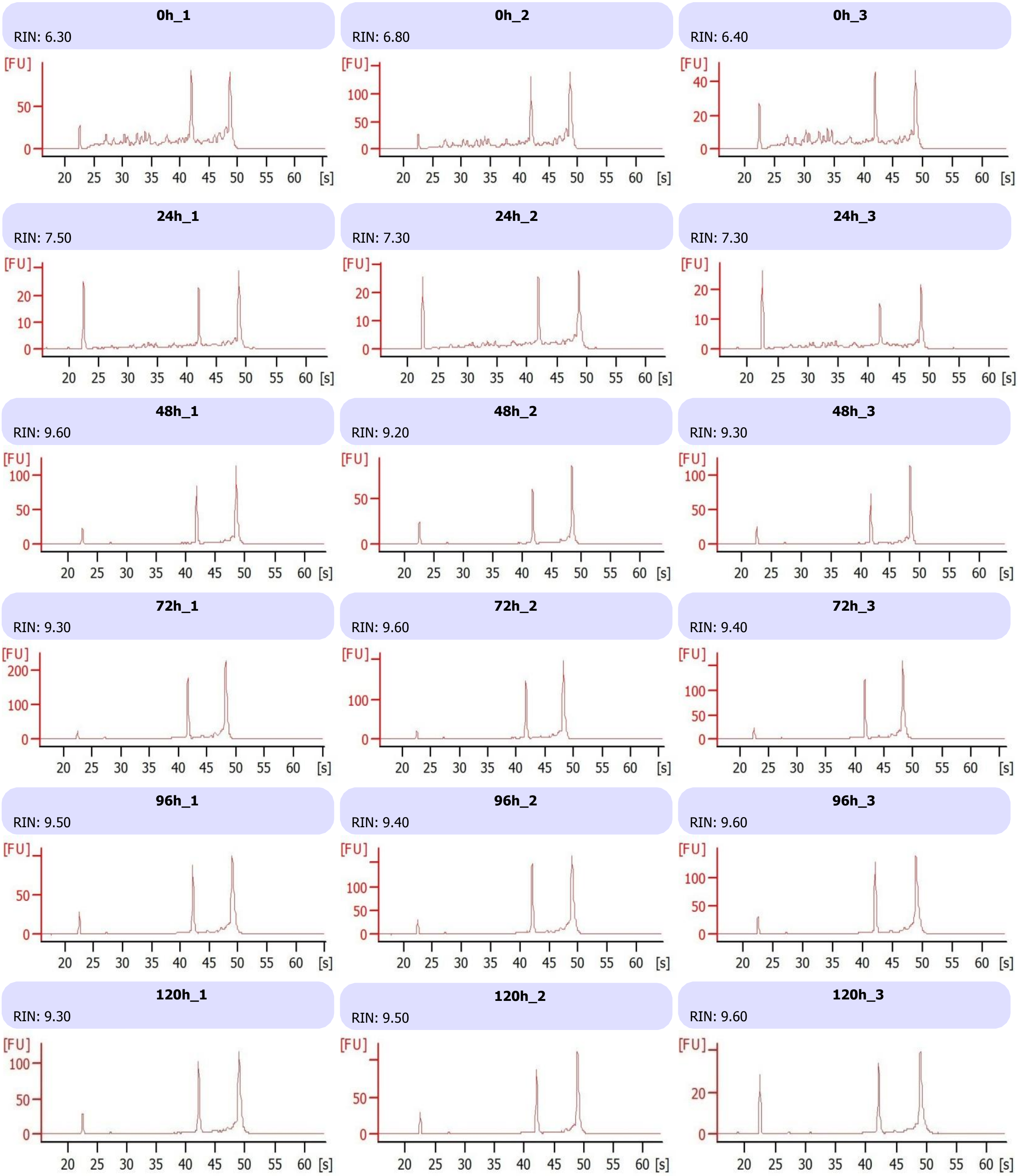

RIN = RNA integrity number.
