# Supplementary material for: Unstable Expression of Commonly Used Reference Genes in Rat Pancreatic Islets Early after Isolation Affects Results of Gene Expression Studies
Source: PLoS One. 2016 Apr 1;11(4):e0152664. doi: 10.1371/journal.pone.0152664 (PMC4817981; doi:10.1371/journal.pone.0152664)
Supplement: S1 Table — Available at http://www.ncbi.nlm.nih.gov/gene/. (PDF) [file pone.0152664.s003.pdf]

S1 Table: Official symbol, ID, full name, function and location of 16 candidate reference genes and the F3 gene. Available at *http://www.ncbi.nlm.nih.gov/gene/.*

Gene	Gene ID	Full Name	Function	Location
Rn18S	100861533	18S ribosomal RNA	Ribosome subunit	Ch14
Actb	81822	Actin, beta	Cytoskeletal protein	Ch12
Arbp	64205	Ribosomal protein, large, P0	Ribosomal protein	Ch12
B2m	24223	Beta-2-microglobulin	Component of MHC class I molecules	Ch3
F3	25584	Coagulation factor III (Tissue factor)	Initiation of blood coagulation	Ch2
Gapdh	24383	Glyceraldehyde-3-phosphate dehydrogenase	Glycolytic enzyme	Ch4
Gusb	24434	Glucuronidase, beta	Glycosaminoglycans degradation	Ch12
Hmbs	25709	Hydroxymethylbilane synthase	Heme biosynthetic pathway	Ch8
Hprt1	24465	Hypoxanthine phosphoribosyltransferase 1	Metabolic salvage of purines	ChX
Pgk1	24644	Phosphoglycerate kinase 1	Glycolytic enzyme	ChX
Ppia	25518	Peptidylprolyl isomerase A	Protein folding	Ch14
Ppib	64367	Peptidylprolyl isomerase B	Protein folding	Ch8
Rplp2	140662	Ribosomal protein, large, P2	Ribosomal protein	Ch1
Tbp	117526	TATA box binding protein	Initiation of gene transcription	Ch1
Tfrc	64678	Transferrin receptor	Cellular iron uptake	Ch11
Ubc	50522	Ubiquitin C	Protein catabolism	Ch12
Ywhaz	25578	Tyrosine 3-monooxygenase/tryptophan 5-monooxygenase activation protein, zeta	Mitochondrial import	Ch7
